# Delineation of the TRAK binding regions of the kinesin-1 motor proteins

**DOI:** 10.1016/j.febslet.2013.09.049

**Published:** 2013-11-29

**Authors:** Thomas S. Randall, Carolyn Moores, F. Anne Stephenson

**Affiliations:** University College London School of Pharmacy, 29/39 Brunswick Square, London WC1N 1AX, United Kingdom

**Keywords:** FEZ1, fasciculation and elongation protein-ζ, FRET, Forster resonance energy transfer, GRIP, glutamate receptor-interacting protein 1, HAP1, Huntingtin-associated protein, HEK, human embryonic kidney, MADD, adaptor protein mitogen-activated protein kinase-activating death domain, TRAK, trafficking kinesin protein, Kinesin, Kinesin adaptor protein, TRAK, Motor protein, Intracellular transport, Mitochondrial trafficking

## Abstract

Understanding specific cargo distribution in differentiated cells is a major challenge. Trafficking kinesin proteins (TRAKs) are kinesin adaptors. They bind the cargo binding domain of kinesin-1 motor proteins forming a link between the motor and their cargoes. To refine the TRAK1/2 binding sites within the kinesin-1 cargo domain, rationally designed C-terminal truncations of KIF5A and KIF5C were generated and their co-association with TRAK1/2 determined by quantitative co-immunoprecipitations following co-expression in mammalian cells. Three contributory regions forming the TRAK2 binding site within KIF5A and KIF5C cargo binding domains were delineated. Differences were found between TRAK1/2 with respect to association with KIF5A.

## Highlights

• TRAK2, a kinesin adaptor protein, binds the cargo binding domain of the kinesin-1 motor, KIF5A.
• Three KIF5A regions were found to contribute to the TRAK2 binding site.
• KIF5A discriminates between TRAK1 and TRAK2 with respect to binding specificity.
• These data yield insights into kinesin/kinesin adaptor protein interactions.

## Structured summary of protein interactions

**TRAK2**physically interacts with **KIF5C** by anti tag coimmunoprecipitation (View interaction)

**TRAK1**physically interacts with **KIF5A** by anti tag coimmunoprecipitation (View interaction)

**TRAK2**physically interacts with **KIF5A** by anti tag coimmunoprecipitation (1, 2)

## 1 Introduction

The kinesins are motor proteins that mediate the transport of organelles and protein complex cargoes within cells. Kinesin-1, the first kinesin to be identified, is a heterotetramer consisting of two heavy chains and two light chains. The heavy chains are comprised of three domains, the motor domain, the stalk domain and the C-terminal cargo binding domain. Although some cargoes associate directly with kinesin via the cargo binding domain or via the light chains, others, particularly those expressed in the central nervous system, associate with kinesin adaptor proteins which then bind to the cargo domain to form trafficking complexes (reviewed in [1]). Known kinesin-1 adaptor proteins include syntabulin, glutamate receptor-interacting protein 1 (GRIP1), UNC76, fasciculation and elongation protein-ζ (FEZ1), adaptor protein mitogen-activated protein kinase-activating death domain (MADD also known as DENN) and the trafficking kinesin (TRAK)/Milton family of proteins (reviewed in [1]). The molecular basis of the interaction between kinesin and the adaptor proteins is not known.

The TRAK family of kinesin adaptor proteins consists of TRAK1 and TRAK2. TRAKs bind to the cargo binding domain of conventional kinesin-1 heavy chains forming a link between the motor protein and cargo [2], [3], [4]. Milton is the *Drosophila* species homologue of TRAK1 and TRAK2 [5]. Huntingtin-associated protein-1 (HAP1) may also be a member of the TRAK family since it has ∼47% homology with both TRAK1 and TRAK2 within a 297 amino acid stretch within the N-terminal domain [6]. HAP1 however has been shown to bind kinesin light chains [7]. The most well established cargo known to be transported by the TRAK/Milton family are mitochondria as a result of the association of TRAK1 and TRAK2 with Miro1 and Miro2, outer mitochondrial membrane, atypical Rho GTPases [8], [9], [10], [11], [12]. The expression of a dominant negative TRAK2 construct that inhibited the binding of kinesin-1 to TRAK1 and TRAK2 in primary cultures of hippocampal neurons, resulted in decreased axonal mitochondrial transport [4]. Similarly, shRNA gene knock-down of TRAK1 also arrested axonal mitochondrial mobility. shRNA gene knock-down of TRAK2 however was without effect [4]. Recently it was found that whereas TRAK1 functions as an adaptor in axons, TRAK2 is requisite for mitochondrial transport in dendrites [12]. This finding thus explains the mismatch between the dominant negative and shRNA findings reported by Stephenson and Brickley [4] and at the same time suggests that there may be differences between the interactions of the two major TRAK family members with kinesin-1 and mitochondrial cargoes.

We have previously shown that TRAK2 co-immunoprecipitates in the brain with the KIF5A kinesin-1 subtype [2]. Using the prototypic kinesin-1, KIF5C, and a combination of co-immunoprecipitations with KIF5C constructs, yeast two-hybrid interaction assays and Förster resonance energy transfer (FRET) studies using yellow and cyan fluorescent protein tagged constructs, we showed that both TRAK1 and TRAK2 bind directly to the KIF5C cargo binding domain [3], [13]. Using a co-immunoprecipitation strategy, we have now extended these studies to refine further the TRAK1 and TRAK2 binding regions within KIF5A, the endogenous neuronal kinesin-1 with which TRAK2 associates, and also KIF5C.

## 2 Materials and methods

### 2.1 Constructs

Mammalian expression KIF5A and KIF5C constructs were generated following PCR amplification of respective parent human cDNAs. KIF5A and KIF5C PCR products were sub-cloned in frame into pcDNA4HisMax (pcDNA) to yield protein products containing an N-terminal His-tag. Final constructs were as follows: pcDNAKIF5A, pcDNAKIF5A_1-961_ (pcDNAKIF5A_Δ962-1032_), pcDNAKIF5A_1-942_ (pcDNAKIF5A_Δ943-1032_), pcDNAKIF5A_1-909_ (pcDNAKIF5A_Δ910-1032_), pcDNAKIF5A_1-885_(pcDNAKIF5A_Δ886-1032_), pcDNAKIF5A_1-883_ (pcDNAKIF5A_Δ884-1032_), pcDNAKIF5A_1-881_ (pcDNAKIF5A_Δ882-1032_), pcDNAKIF5A_1-879_ (pcDNAKIF5A_Δ880-1032_), pcDNAKIF5A_1-877_ (pcDNAKIF5A_Δ878-1032_), pcDNAKIF5A_1-861_ (pcDNAKIF5A_Δ862-1032_), pcDNAKIF5A_1-825_ (pcDNAKIF5A_Δ826-1032_), pcDNAKIF5C, pcDNAKIF5C_1-889_ (pcDNAKIF5C_Δ890-957_), pcDNAKIF5C_1-881_ (pcDNAKIF5C_Δ882-957_) and pcDNAKIF5C_1-828_ (pcDNAKIF5C_Δ829-957_). Deletion mutagenesis by overlap extension PCR was used to generate pcDNAKIF5A_Δ877-883_ (i.e. amino acids 877–883, have been deleted). pCMVTRAK2 (rat) was generated as in [6]; recombinant TRAK2 has a C-terminal Flag-tag. pCMVTRAK1 (human) was as in [2]; recombinant TRAK1 has a C-terminal c-Myc tag.

### 2.2 Antibodies

Primary anti-His-tag antibodies conjugated to horseradish peroxidase were from Sigma–Aldrich (Gillingham, Dorset, UK); anti-FLAG antibodies were as in [6]; anti-c-Myc antibodies were from Thermo Fisher Scientific (Massachusetts, USA). Rabbit and mouse horseradish peroxidase-linked secondary antibodies were from Amersham Biosciences (UK).

### 2.3 Mammalian cell transfections, immunoprecipitations and immunoblotting

Human embryonic kidney (HEK) 293 cells were transiently transfected with pCMVTRAK2 or pCMVTRAK1 with either pcDNAKIF5A, pcDNAKIF5A truncations, pcDNAKIF5A_Δ877-883_, pcDNAKIF5C or pcDNAKIF5C truncation constructs in equal ratios by the calcium phosphate method. Cell homogenates were collected 48 h post-transfection. Triton X-100 (1% v/v) extracts were prepared and centrifuged at 100,000×*g* to yield solubilised extracts of transfected HEK 293 cells. Immunoprecipitations were carried out using either anti-FLAG, anti-c-Myc antibodies or non-immune Ig. Immune pellets were precipitated by the addition of protein A Sepharose and analysed by immunoblotting using anti-FLAG, anti-c-Myc and anti-His antibodies as appropriate as previously described (e.g. [14]). Note that for all immunoblots, 10% of the immune and non-immune pellets were probed with the precipitating antibody whereas 90% of the immune and non-immune pellets were probed with the appropriate antibodies that were used to determine co-precipitation. Immunoblots were quantified by band densitometry analysis using GeneTools™ software. Statistical analysis was carried out using a paired students *t*-test and significance given as ^∗^*P* < 0.05, ^∗∗^*P* < 0.01 and ^∗∗∗^*P* < 0.001.

## 3 Results and discussion

### 3.1 TRAK2 co-immunoprecipitates with the KIF5A cargo binding domain

**Fig. 1:**
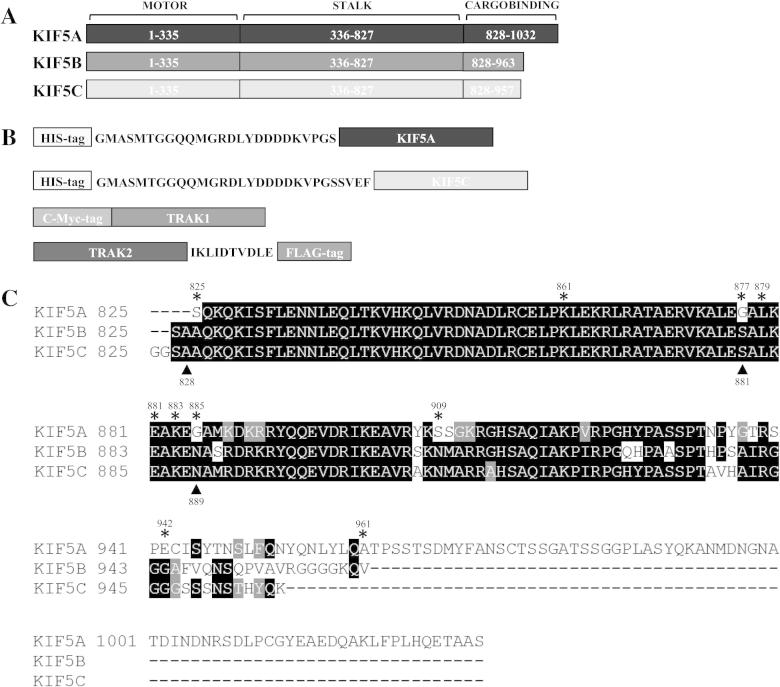
Schematic diagram and amino acid sequence alignment of KIF5A, KIF5B and KIF5C kinesin-1 subtypes. (A) Schematic depicting the motor, stalk and cargo binding domains of the kinesin-1 family members, KIF5A, KIF5B and KIF5C. (B) Schematic representations of the epitope-tagged KIF5A, KIF5C, TRAK1 and TRAK2 proteins used in this study. (C) An alignment of the amino acid sequences of the human KIF5A (Protein accession number: NP004975), KIF5B (Protein accession number: NP004512) and KIF5C (Protein accession number: NP004513) cargo binding domains. Identical amino acids are highlighted in black; amino acid similarities are highlighted in grey. ^∗^ and ▴ show the positions of KIF5A and KIF5C truncations respectively.

**Fig. 2:**
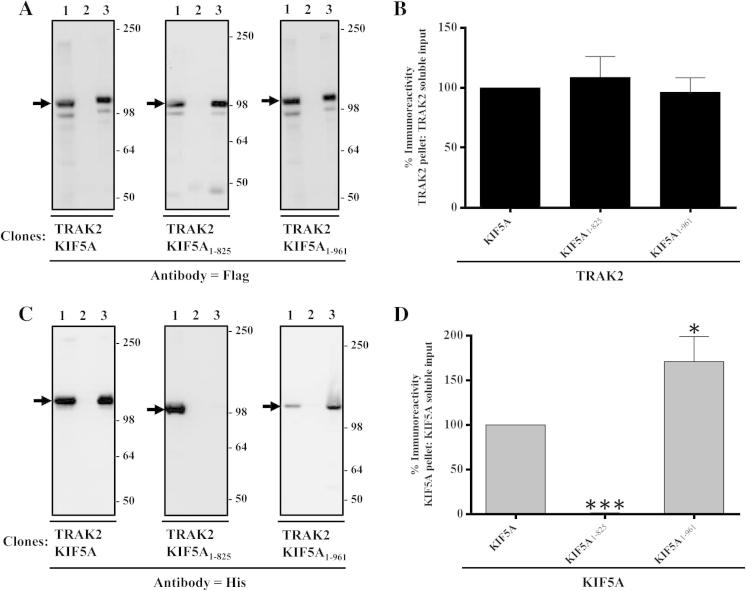
Association of TRAK2 with KIF5A, KIF5A_1-825_ and KIF5A_1-961_: demonstration by co-immunoprecipitation. HEK 293 cells were co-transfected with pCMVTRAK2 and either pcDNAKIF5A, pcDNAKIF5A_1-825_ or pcDNAKIF5A_1-961_. Cell homogenates were prepared 48 h post-transfection, detergent solubilised and co-immunoprecipitations carried out using either anti-FLAG antibodies or non-immune Ig. (A and C) Representative immunoblots of immune pellets. For this and all subsequent immunoblots, lane 1 = transfected HEK 293 detergent-solubilized cell homogenates; lane 2 = non-immune pellet and lane 3 = anti-FLAG pellet. →, the position of immunoreactive bands. The positions of molecular mass standards (kDa) are shown on the right*.* (B and D) Histograms showing the quantitative analyses of co-immunoprecipitations. The ratios of immunoreactivities of the respective immune pellets to detergent solubilized input were determined and expressed as percentages of the control (i.e. wild-type TRAK2^FLAG^/KIF5A^HIS^ transfectants = 100%). Immunoblots are representative of at least n = 3 co-immunoprecipitations from at least *n* = 3 independent transfections. (B and D Means ± S.E.M. for at least *n* = 3 co-immunoprecipitations from at least *n* = 3 independent transfections. ^∗^*P* < 0.05; ^∗∗∗^*P* < 0.001.

TRAK2 was shown previously to associate directly with the KIF5C cargo binding domain [3] but, since TRAK2 associates with KIF5A in native tissue, in the first instance it was investigated if TRAK2 also binds to the KIF5A cargo binding domain. A comparison of the three kinesin-1 isoforms, KIF5A, KIF5B and KIF5C are shown in Fig. 1 together with the tagged constructs generated. Thus, KIF5A was truncated at 825 which resulted in the deletion of the C-terminal cargo binding domain generating an N-terminally His-tagged construct, KIF5A_1-825_. This was co-expressed with C-terminal FLAG-tagged TRAK2 in HEK 293 cells in parallel with wild-type TRAK2/KIF5A co-transfectants. Transfected cells were harvested, solubilized, immunoprecipitated with anti-FLAG or non-immune Ig and immune precipitates were analysed by quantitative immunoblotting. The results are shown in Fig. 2. For both transfection conditions, anti-FLAG but not control non-immune antibodies immunoprecipitated a major molecular weight species of M_r_ ∼ 110 kDa consistent with the size of TRAK2 (Fig. 2A). Immunoblotting with anti-His antibodies revealed the presence of KIF5A, M_r_ ∼ 100 kDa in immunoprecipitates from wild-type TRAK2^FLAG^/KIF5A^His^ transfectants. However, deletion of the C-terminal cargo binding domain resulted in the loss of anti-His immunoreactivity in immune pellets (Fig. 2C). Thus similarly to KIF5C, TRAK2 binds to the KIF5A cargo binding domain.

KIF5A has an extended C-terminal region compared to KIF5B and KIF5C (Fig. 1). To determine the significance of this region in terms of TRAK2 binding, this was deleted to generate KIF5A_1-961_ and transfections and immunoprecipitations were carried out as above always in parallel with wild-type TRAK2^FLAG^ and KIF5A^His^. Deletion of KIF5A 962–1032 amino acids had no qualitative effect on the co-immunoprecipitation of KIF5A_1-961_ with TRAK2 compared to wild-type (Fig. 2C). However, quantitative analyses of the co-immunoprecipitations revealed that removal of the extended KIF5A tail resulted in an increased (∼70%) efficiency of TRAK2/KIF5A_1-961_ association. This implies that the KIF5A tail may be important in regulating the efficacy of TRAK2/KIF5A association. Note that the efficiency of TRAK2 immunoprecipitation is not affected by the co-expression of the different KIF5A constructs (Fig. 2B).

### 3.2 Refinement of the TRAK2 binding region of KIF5A

**Fig. 3:**
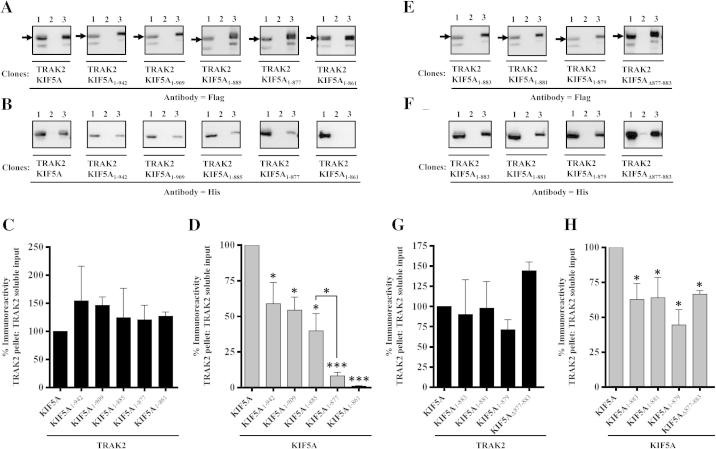
Refinement of the KIF5A TRAK2 binding site. HEK 293 cells were co-transfected with pCMVTRAK2 and either pcDNAKIF5A, pcDNAKIF5A_1-942_, pcDNAKIF5A_1-909_, pcDNAKIF5A_1-885_, pcDNAKIF5A_1-861_ (A–D) or pcDNAKIF5A, pcDNAKIF5A_1-883_, pcDNAKIF5A_1-881_, pcDNAKIF5A_1-879_, pcDNA_Δ877-883_ (E and F). Cell homogenates were prepared 48 h post-transfection, detergent solubilised and immunoprecipitations carried out using either anti-FLAG antibodies or non-immune Ig. A, B, E and F are representative immunoblots of the immune pellets with gel lanes as in Fig. 2. →, the position of immunoreactive bands. C, D, G and H, histograms showing the quantitative analyses of co-immunoprecipitations. The ratios of immunoreactivities of the respective immune pellets to detergent solubilized input were determined and expressed as percentages of the control (i.e. wild-type TRAK2^FLAG^/KIF5A^HIS^ transfectants = 100%). Immunoblots are representative of at least *n* = 3 co-immunoprecipitations from at least *n* = 3 independent transfections. C, D, G and H D, means ± S.E.M. for at least *n* = 3 co-immunoprecipitations from at least *n* = 3 independent transfections. ^∗^*P* < 0.05; ^∗∗∗^*P* < 0.001.

**Fig. 4:**
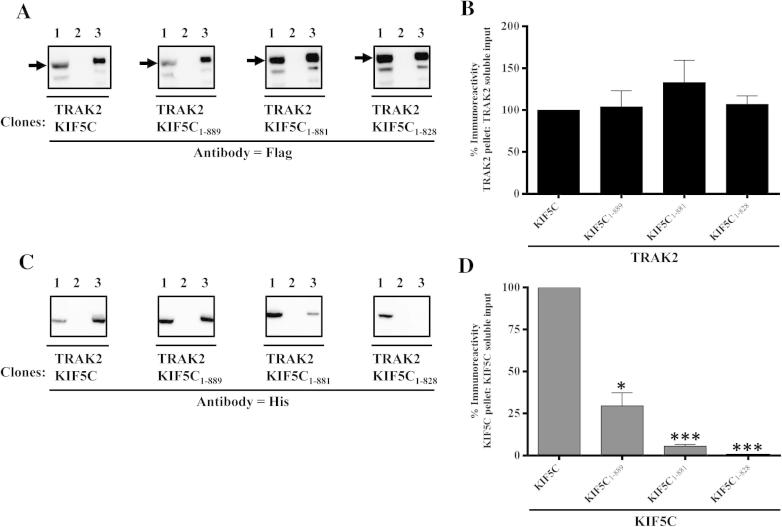
Association of TRAK2 with KIF5C: demonstration by co-immunoprecipitation. HEK 293 cells were co-transfected with pCMVTRAK2 and either pcDNAKIF5C, pcDNAKIF5C_1-889_, pcDNAKIF5C_1-881_ or pcDNAKIF5C_1-828_. Cell homogenates were prepared 48 h post-transfection, detergent solubilised and immunoprecipitations carried out using either anti-FLAG antibodies or non-immune Ig. (A and C) Representative immunoblots of the immune pellets with gel lanes as in Fig. 2. →, the position of immunoreactive bands. (B and D) Histograms showing the quantitative analyses of co-immunoprecipitations. The ratios of immunoreactivities of the respective immune pellets to detergent solubilized input were determined and expressed as percentages of the control (i.e. wild-type TRAK2^FLAG^/KIF5C^HIS^ transfectants = 100%). Immunoblots are representative of at least *n* = 3 co-immunoprecipitations from at least *n* = 3 independent transfections. ^∗^*P* < 0.05; ^∗∗∗^*P* < 0.001.

**Fig. 5:**
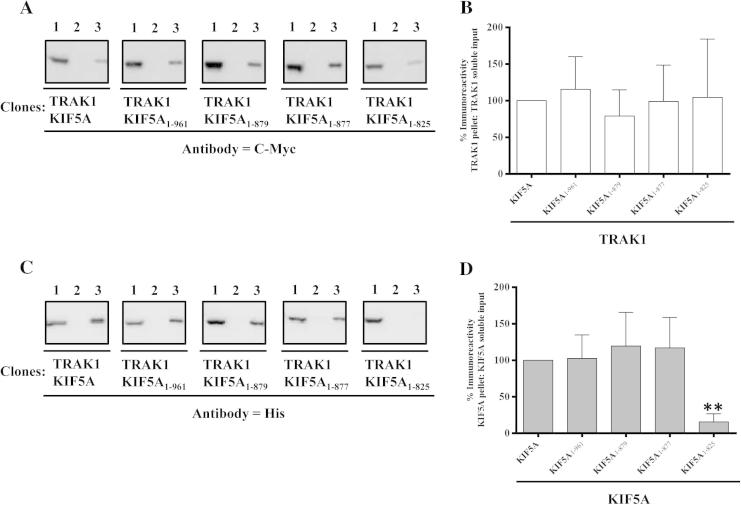
Association of TRAK1 with KIF5A: demonstration by co-immunoprecipitation. HEK 293 cells were transfected with pCMVTRAK1 and either pcDNAKIF5A, pcDNAKIF5A_1-961_, pcDNAKIF5A_1-879_, pcDNAKIF5A_1-877_ or pcDNAKIF5A_1-825_. Cell homogenates were prepared 48 h post-transfection, detergent solubilised and immunoprecipitations carried out using either anti-c-Myc antibodies or non-immune Ig. (A and C) Representative immunoblots of the immune pellets with gel lanes as in Fig. 2. →, the position of immunoreactive bands. (B and D) Histograms showing the quantitative analyses of co-immunoprecipitations. The ratios of immunoreactivities of the respective immune pellets to detergent solubilized input were determined and expressed as percentages of the control (i.e. wild-type TRAK1^C-Myc^/KIF5A^HIS^ transfectants = 100%). Immunoblots are representative of at least *n* = 3 co-immunoprecipitations from at least *n* = 3 independent transfections. (B and D) Means ± S.E.M. for at least *n* = 3 co-immunoprecipitations from at least *n* = 3 independent transfections. ^∗∗^*P* < 0.01.

To refine the TRAK2 binding site within the KIF5A cargo binding domain, a series of KIF5A truncations were generated. Each was co-expressed with TRAK2 and immunoprecipitation assays carried out as above to determine if the truncated constructs retained the ability to co-immunoprecipitate with TRAK2. The rationale for the site of the KIF5A truncations was based on the amino acid sequence similarities and differences between the three kinesin-1 isoforms. The C-terminal region ∼20 amino acids of KIF5B and KIF5C and the corresponding aligned region of KIF5A is variable thus KIF5A_1-942_ was designed to delete this region. KIF5A_1-942_ retained the ability to co-immunoprecipitate with TRAK2 albeit with a reduced efficiency; the percentage KIF5A_1-942_ co-immunoprecipitated with TRAK2 was significantly reduced, ∼40%, compared to wild-type TRAK2/KIF5A transfections (Fig. 3D). Further truncations at KIF5A 909 and KIF5A 885 resulted in no additional decrease in the co-immunoprecipitation efficiency compared to KIF5A 942 truncations. Truncation at KIF5A 861 however resulted in the loss of co-immunoprecipitation with TRAK2 implying that the highly conserved region KIF5A numbering 825–861, does not contribute to the binding of TRAK2 (Fig. 3D). It was notable that a small but significant co-immunoprecipitation (∼10%) was detected for constructs truncated at KIF5A 877; this was statistically significantly different to the percentage KIF5A co-immunoprepicipated with TRAK2 for constructs KIF5A_1-942_; KIF5A_1-909_ and KIF5A_1-885_ (Fig. 3D). Thus using this approach, three regions within the KIF5A C-terminal region were shown to contribute to the TRAK2 binding site i.e. KIF5A 942–961; KIF5A 877–885 and KIF5A 861–877. (Note that full immunoblots for Fig. 3, Fig. 4, Fig. 5 can be viewed in the Supplementary Information, Figs. S1–S3).

The region KIF5A 877–883 was studied in more detail. Three additional truncations were made at KIF5A 883, KIF5A 881 and KIF5A 879 plus the region 877–883 was deleted. The constructs truncated at KIF5A 883, KIF5A 881 and KIF5A 879 co-immunoprecipitated with TRAK2 with a similar efficiency to KIF5A_1-885_ as indeed did the KIF5A_Δ877-883_ deletion. The latter was unexpected since this region, amino acid sequence GALKEAKE, had been clearly implicated in the efficiency of KIF5A/TRAK2 association (Fig. 3D). It may be explained by an alteration of the KIF5A tertiary structure such that other amino acids requisite for the binding of TRAK2 are now available to compensate for the deleted region thus the predicted loss in efficacy of co-immunoprecipitation is circumvented. In the context of the full length KIF5A therefore, the other sites of interaction are sufficient to support partial association with TRAK2 even when 877–883 is removed.

### 3.3 Are the TRAK2 binding sites of KIF5A conserved between KIF5A and KIF5C?

To determine if there are any differences in the interaction of TRAK2 between KIF5A and KIF5C, similar experiments were carried out using KIF5C constructs. We have previously shown that the KIF5C non-motor domain co-immunoprecipitated with TRAK2 [3] suggesting that TRAK2 bound to the KIF5C cargo binding domain. Here, we deleted the KIF5C cargo binding domain and found that KIF5C_1-828_ lost the ability to be co-immunoprecipitated by TRAK2 (Fig. 4). Thus these deletion construct experiments are in agreement with those employing isolated domains, i.e. TRAK2 does indeed bind to the KIF5C cargo binding region. Two additional KIF5C truncations were generated KIF5C_1-881_ which corresponds to KIF5A_1-877_ and KIF5C_1-889_ which aligns with KIF5A_1-885_ (Fig. 1). A similar overall profile was obtained. TRAK2 co-immunoprecipitated KIF5C_1-881_ with an efficiency of ∼10% compared to wild-type KIF5C; the efficiency of KIF5C_1-889_ co-immunoprecipitation by TRAK2 was also reduced. No significant difference in immunoprecipitation efficiencies between KIF5A_1-885_ and KIF5C_1-881_ was detected. Values for the decreased efficiency of co-immunoprecipitation were: 70.1 ± 12.3% (KIF5C_1-881_) and 60.2 ± 12.3% (KIF5A_1-885_); *P* < 0.01.Thus the TRAK2 binding sites of KIF5A and KIF5C are similar.

### 3.4 KIF5A discriminates between TRAK1 and TRAK2 with respect to binding specificity

Differences between the interaction of TRAK1 and TRAK2 with kinesin motor proteins were recently reported [12]. It was thus of relevance to determine if TRAK1 had a similar binding profile to that of TRAK2 for the KIF5A truncations that have been studied here. Thus an N-terminally c-Myc tagged-TRAK1 was co-expressed with KIF5A_1-961_, KIF5A_1-879_, KIF5A_1-877_ and KIF5A_1-825_ in parallel with wild-type TRAK1/KIF5A combinations; co-immunoprecipitations were carried out as before except that anti-c-Myc antibodies were used as the primary precipitating antibody. The results are shown in Fig. 5. Interestingly, the TRAK1 co-immunoprecipitation profile was in marked contrast to that observed for TRAK2. Removal of the extended C-terminal KIF5A tail did not result in enhanced co-immunoprecipitation. TRAK1 retained the ability to co-immunoprecipitate KIF5A in the absence of the cargo binding domain albeit with a reduced efficiency of 15 ± 11% (*n* = 3). Further, no decrease in co-immunoprecipitation efficiency was evident for the KIF5A_1-881_ and KIF5A_1-877_ truncations. Note that as for the TRAK2 immunoprecipitation assays, the efficiency of TRAK1 immunoprecipitations is not affected by the co-expression of the different KIF5A constructs (Fig. 5).

### 3.5 Conclusions

**Fig. 6:**
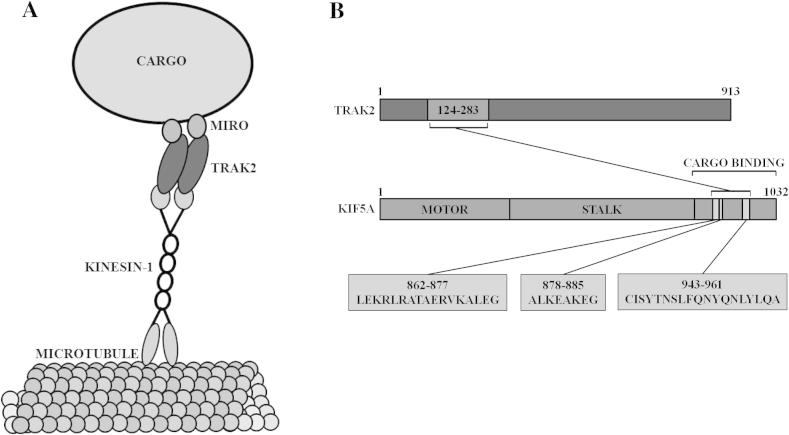
A schematic diagram depicting the association between TRAK2 and kinesin-1. (A) Is a schematic diagram of the kinesin-1/TRAK/Miro mitochondrial trafficking complex. (B) Illustrates the three KIF5A regions that were identified in this paper as contributing to the TRAK2 binding domain.

In this paper, we have refined the TRAK2 binding site within the kinesin-1 cargo binding domain of the molecular motor, KIF5A. We have shown that there are three contributing regions of KIF5A to the binding site for TRAK2. Our data show that, as these regions are sequentially truncated from the motor’s C-terminal end, the interaction between the motor and its adaptor is gradually reduced. However, deletion of an exemplar individual region (KIF5A 877–883) only modestly reduced the interaction. This supports the idea that the KIF5A–TRAK2 interaction is multivalent and could act to ensure stable motor-cargo interaction during intracellular trafficking; dimerization of both motor and adaptor molecules further enhances this stability (Fig. 6). A similar multivalent profile was found for the TRAK2 binding site within the kinesin-1 isoform, KIF5C. We also observed that the unique KIF5A C-terminal sequence was not required for TRAK binding. In fact, removal of this region enhanced the interaction between KIF5A and TRAK2 suggesting that isoform-specific auto-inhibition may provide a further layer of transport regulation by neuronal kinesins.

Interestingly, the KIF5A TRAK1 binding site was shown to be distinct to that of TRAK2 suggesting a differential interaction between these two kinesin adaptor proteins and molecular motors which may impact in the determination and selectivity of cargo binding and delivery within neurons. In fact, we had previously found that TRAK1 behaved differently to TRAK2 in terms of association with kinesin-1 isoforms [2]. TRAK1 and TRAK2 both co-immunoprecipitated with endogenous KIF5B and exogenous KIF5C following expression in HEK 293 cells. In yeast two-hybrid interaction assays however, TRAK1 fish constructs did not result in reporter gene activity following co-transformation with a KIF5C bait. This was in contrast to TRAK2/KIF5C bait/fish combinations where robust reporter gene expression was evident for both full length TRAK2 and TRAK2_124-283_, the kinesin-1 binding domain [2]. The equivalent TRAK1 region, TRAK1_124-283_, to the TRAK2_124-283_ kinesin-1 binding domain, did not co-immunoprecipitate with full length KIF5C again in contrast to TRAK2_124-283_/KIF5C co-expression and co-immunoprecipitation. This suggests that TRAK1 may adopt different conformational states that lead to the observed perplexing inconsistencies. Interestingly, van Spronsen et al. [12] also reported differences between TRAK1 and TRAK2 in terms of their respective association with kinesin-1. In contrast to Brickley et al. [2], they found that TRAK2 did not associate with endogenous KIF5B in HEK 293 cells. Furthermore, only TRAK1 was shown to have a strong interaction with kinesin-1 in rat brain [12]. These findings were explained by FRET acceptor photobleaching experiments which suggested that TRAK2 forms a stable head to tail dimer whereas TRAK1 has a more dynamic and transient conformation. These conformational differences were speculated to contribute to different cargo binding capabilities and targeting i.e. TRAK1 mediates mitochondrial transport in axons, whereas TRAK2 is the major adaptor in dendrites [4], [12]. The discrepancies between the two groups are yet to be explained.

Recently, Chen and Sheng [15] reported that syntaphilin, a protein shown to anchor axonal mitochondria, competes with TRAK2 for the binding to kinesin-1 (KIF5C). They further reported that co-expression of TRAK2 with microtubules and KIF5C resulted in an enhanced KIF5 motor ATPase activity [15]. The latter observation suggests that the binding of TRAK2 within the cargo binding region influences the N-terminal motor activity of kinesin-1 presumably via conformational changes. The competition between syntaphilin and TRAK2 for the binding to kinesin implies that at least in part, they share a common KIF5 binding site. It would be interesting to test the ability of the KIF5A truncations generated here for their ability to co-immunoprecipitate syntaphilin.

The structure of the kinesin non-motor domain has so far been experimentally intractable presumably in part because of difficulties in crystallization explained perhaps by intrinsic flexibility [16]. However, a complex between kinesin-1 motor dimer and an auto-inhibitory portion of the kinesin-1 tail domain has been structurally characterised, providing insight into the molecular basis of kinesin transport efficiency [17]. Strikingly, the motor tail peptide responsible for blockage of motor activity (KIF5B 937–952, including the well-characterised and completely conserved IAK sequence [18], [19] is located in between two of the sites of kinesin-TRAK interaction that we have identified. Thus, an auto-inhibited kinesin molecule would not be expected to be accessible for TRAK binding or available for transport, whereas binding to a TRAK adaptor would be expected to activate kinesin motility.

The studies described and cited herein support functional cross-talk between the motor and cargo binding non-motor domains and they identify three regions that are important for at least one family of kinesin adaptor protein, the TRAKs. It remains to be established if the same determinants mediate the binding of other kinesin adaptors (including syntaphilin) or, alternatively, they bind to other regions. This could provide a molecular basis for the discrimination of kinesin/kinesin adaptor protein/cargo specificity ultimately defining mechanisms of differential intracellular transport.
