# Supplementary material for: Delineation of the TRAK binding regions of the kinesin-1 motor proteins
Source: FEBS Lett. 2013 Nov 29;587(23):3763–9. doi: 10.1016/j.febslet.2013.09.049 (PMC3853714; doi:10.1016/j.febslet.2013.09.049)

Supplementary Information

Full length immunoblots for Figures 3, 4 and 5 in the main body of the text.

Figure S1: Refinement of the KIF5A TRAK2 binding site

HEK 293 cells were co-transfected with pCMVTRAK2 and either pcDNAKIF5A, pcDNAKIF5A$_{1\text{-}942}$, pcDNAKIF5A$_{1\text{-}909}$, pcDNAKIF5A$_{1\text{-}885}$, pcDNAKIF5A$_{1\text{-}861}$ (A-B) or pcDNAKIF5A, pcDNAKIF5A$_{1\text{-}883}$, pcDNAKIF5A$_{1\text{-}881}$, pcDNAKIF5A$_{1\text{-}879}$, pcDNA$_{\Delta 877\text{-}883}$ (C-D). Cell homogenates were prepared 48 h post-transfection, detergent solubilised and immunoprecipitations carried out using either anti-FLAG antibodies or non-immune Ig. A, B , C and D are immunoblots of the immune pellets with gel lanes as in Figure 2. →, the position of immunoreactive bands.

Figure S2: Association of TRAK2 with KIF5C: demonstration by co-immunoprecipitation

HEK 293 cells were co-transfected with pCMVTRAK2 and either pcDNAKIF5C, pcDNAKIF5C$_{1\text{-}889}$, pcDNAKIF5C$_{1\text{-}881}$ or pcDNAKIF5C $_{1\text{-}828}$. Cell homogenates were prepared 48 h post-transfection, detergent solubilised and immunoprecipitations carried out using either anti-FLAG antibodies or non-immune Ig. A and B, immunoblots of the immune pellets with gel lanes as in Figure 2. →, the position of immunoreactive bands.

Figure S3: Association of TRAK1 with KIF5A: demonstration by co-immunoprecipitation

HEK 293 cells were transfected with pCMVTRAK1 and either pcDNAKIF5A, pcDNAKIF5A$_{1\text{-}961}$, pcDNAKIF5A$_{1\text{-}879}$, pcDNAKIF5A$_{1\text{-}877}$ or pcDNAKIF5A$_{1\text{-}825}$. Cell homogenates were prepared 48 h post-transfection, detergent solubilised and immunoprecipitations carried out using either anti–c-Myc antibodies or non-immune Ig. A and B, immunoblots of the immune pellets with gel lanes as in Figure 2. →, the position of immunoreactive bands.

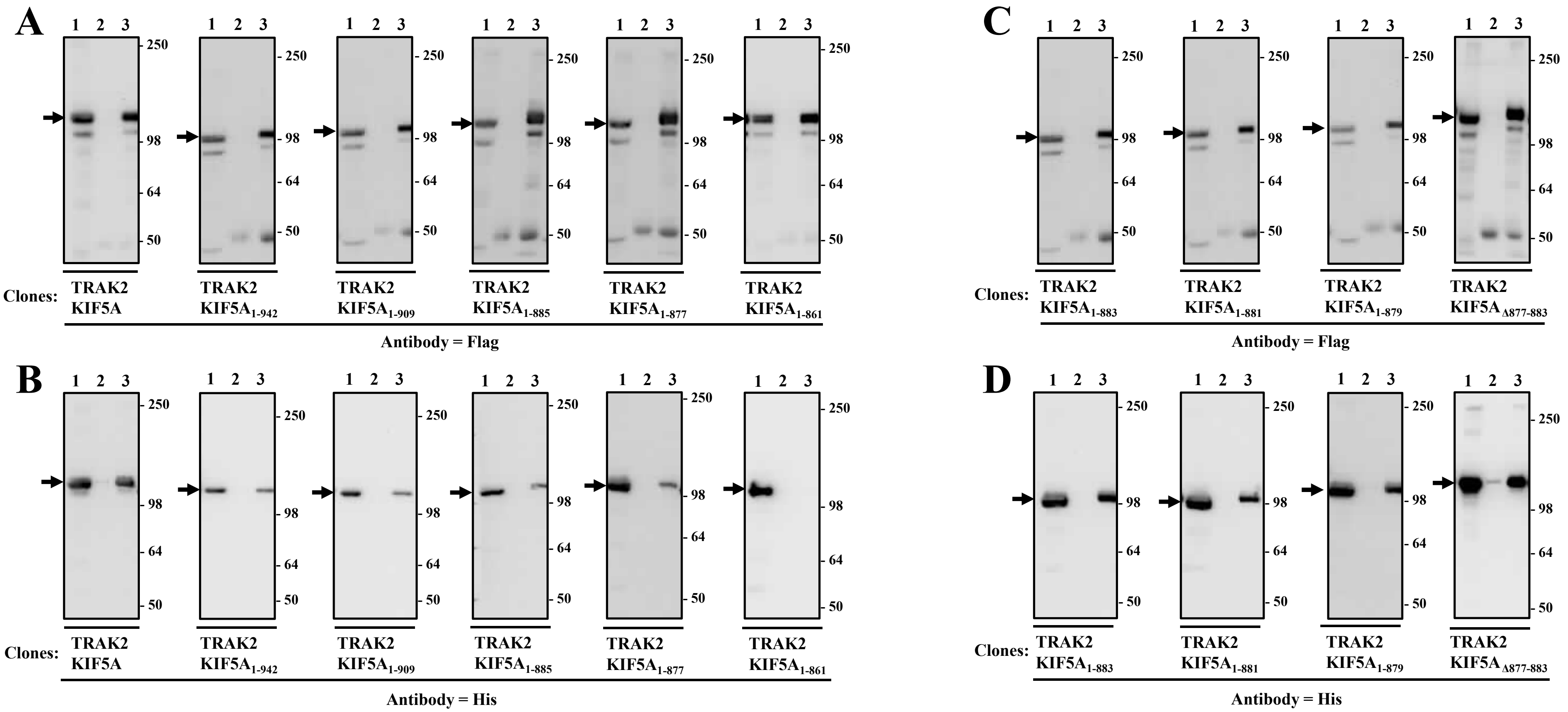

Supplementary Figure 1

Supplementary Figure 2

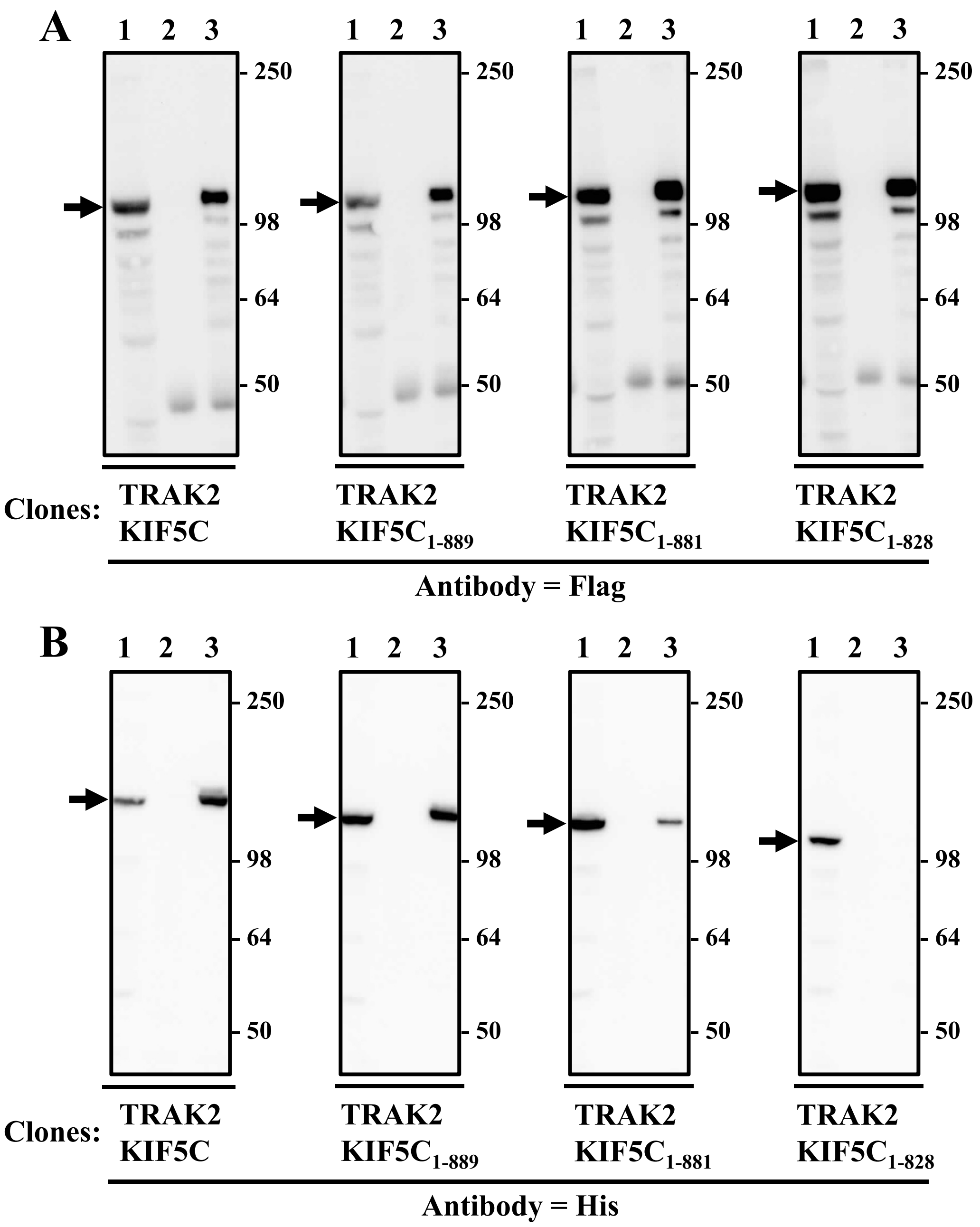

Supplementary Figure 3

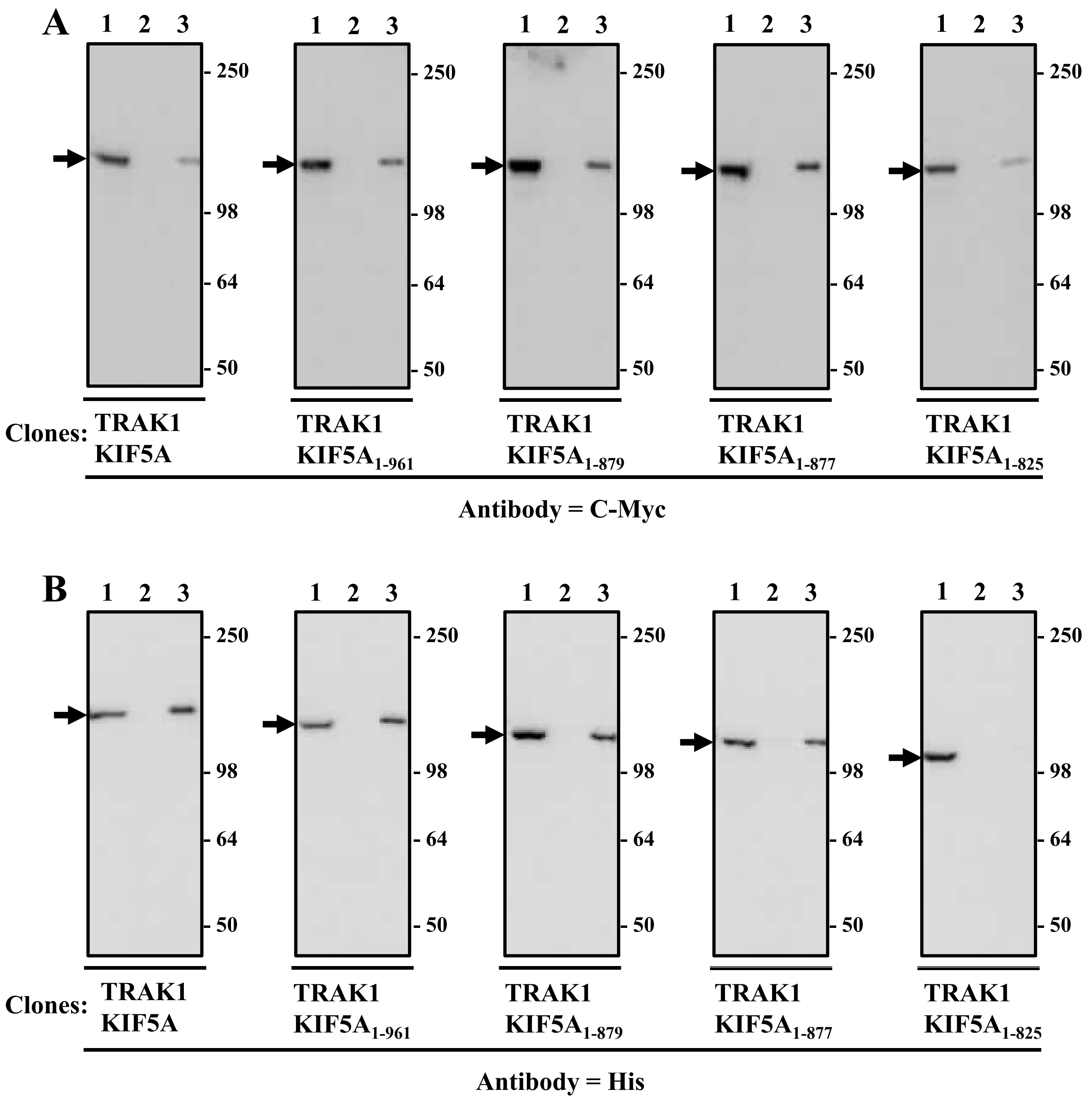

Supplement: Supplementary data 1 — Supplementary Figures. [file mmc1.pdf]
